# Supplementary material for: Sub-volt high-speed silicon MOSCAP microring modulator driven by high-mobility conductive oxide
Source: Nat Commun. 2024 Jan 27;15:826. doi: 10.1038/s41467-024-45130-4 (PMC10821938; doi:10.1038/s41467-024-45130-4)
Supplement: Supplementary file 1 — Supplementary Information [file 41467_2024_45130_MOESM1_ESM.pdf]

Sub-Volt High-Speed Silicon MOSCAP Microring Modulator Driven by High Mobility Conductive Oxide (Supplementary Information)

Wei-Che Hsu[1,2], Nabila Nujhat[1], Benjamin Kupp[1], John F. Conley, Jr[1], Haisheng Rong[3], Ranjeet Kumar[3], and Alan X. Wang[1,2,*]

[1]School of Electrical Engineering and Computer Science, Oregon State University, Corvallis, Oregon 97331, USA

[2]Department of Electrical and Computer Engineering, Baylor University, One Bear Place #97356, Waco, Texas 76798, USA

[3]Intel Corporation, 3600 Juliette Ln, Santa Clara, CA 95054, USA

[*]alan_wang@baylor.edu

I. Review of High-Speed Si Microring Modulators Driven by Various Structures

Table S1 summarizes published state-of-the-art Si microring modulators (Si-MRM). Typically, the quality factor (Q-factor) of Si-MRM is designed below 5000 to achieve an optical bandwidth above 40 GHz. Most Si-MRMs require a high driving voltage (V_{pp}) for electro-optic (E-O) modulation due to their low E-O efficiency. Higher insertion loss (IL) results in a sharper resonance. Therefore, some existing Si-MRMs sacrifice the IL to lower the driving voltage. Integrating the plasmonic/E-O polymer phase shifter into the Si-MRM achieved exceptionally high E-O efficiency. However, the plasmonic structure is inherently lossy and reduces the Q-factor of the Si-MRM. Our work on titanium-doped indium oxide (ITiO)-gated MOSCAP Si-MRM not only offers enhanced E-O-efficiency but also maintains a sufficient Q-factor to achieve a low driving voltage and support a large optical bandwidth.

Table S1: Comparison of performance of the high-speed MRM.

Modulator structure	Operation band	Q-factor	E-O efficiency (pm/V)	V_{pp} (V)	$V_\pi L$ (V•cm)	$V_\pi L \times \alpha$* (V•dB)	Energy efficiency (fJ/bit)	IL (dB)	f_{3dB} (GHz)	Data rate (Gb/s)	Ref.
Reversed/Forward PN junction (Microdisk)	L	~6350	250	0.5	~0.098	6.79	1	1	21	25	1
Reversed PN junction	O	4200	~40	0.8 (~2)	0.53	53.18	5.3	9 (~3)	58	128	2
Reversed PN junction	O	4500	26.4	3	0.825	75.19	~85.5	3	>60	120	3

MOSCAP-Si	C	3500	130	1.5	0.24	23.23	180	3	1.7	3	4
MOSCAP-Si	O	~4600	~170	1	~0.13	11.42	5	1.3	2	10	5
MOSCAP-Si	C	4600	40	1.6	0.7	57.41	~24.32	9	50	112	6
MOSCAP-III-V	O	8143	~20	4	1	54.86	~250	2	15	28	7
MOSCAP-graphene	C	3396	~33	6	-	93.10	21	5.8	52	40	8
Plasmonic-Polymer	C	700	178	4	0.015	75	12.3	4	176	220	9
MOSCAP-ITiO	O	4600	117	0.8	0.12	16.68	53	3	11	25	This work

~ Didn't mention it in the article. Measured from spectra or calculated based on other parameters in the articles.
- Not available in the article
* Estimated from Q-factor, E-O efficiency, and $V_{\pi}L$

II. Optimizing Radius for ITiO-Gated MOSCAP Si-MRM

The ITiO-gated MOSCAP Si-MRM is designed to achieve a Q-factor ranging from 5000 to 6000 that ensures sufficient optical bandwidth while minimizing the driving voltage. Once the ITiO is deposited on the Si ring waveguide, it introduces optical absorption losses that can decrease the Q-factor. To maintain the Q-factor after ITiO deposition, it is crucial to ensure that the loss from the Si ring waveguide is negligible compared to the optical absorption from ITiO. In other words, the Q-factor of the passive Si microring resonator should be significantly higher than 5000, for instance, around 20000. To determine the appropriate radius for the design, Fig. S1 presents the simulated loss of the bent passive Si waveguide at various radii. The geometry and doping profiles of the Si waveguide remain consistent with the design illustrated in Fig. 1. Since the waveguide is designed to be 300 nm wide, it exhibits significant bending losses at small radii. As the radius increases, the loss decreases accordingly. It is observed that when the radius exceeds 8 μm, the loss of the passive Si microring becomes sufficiently low to support a Q-factor greater than 20000. Based on the simulation results, the radius of 8 μm is chosen for the ITiO-gated MOSCAP Si-MRM design. It ensures that the passive Si microring resonator maintains a high Q-factor, even after the deposition of ITiO, enabling the desired performance.

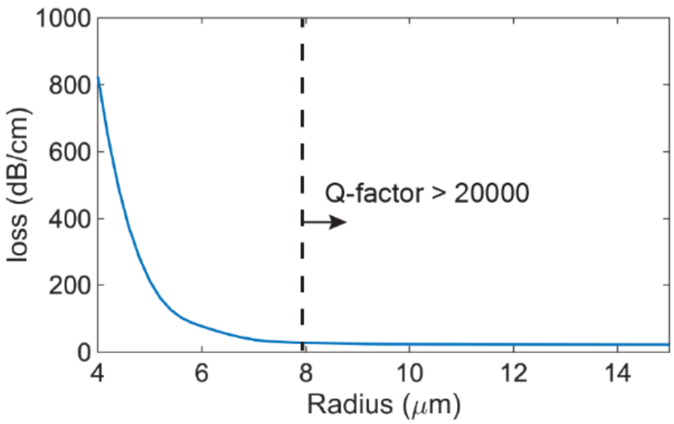

Fig. S1: Simulated optical loss of bent Si waveguide at various radii.

III. ITiO Thin Film Characterization

The ITiO material was characterized by RF-sputtering it onto lime glass in the same batch as the device. The sputtering process maintained consistent conditions, including a high substrate heating temperature of 500 °C. The thickness of the ITiO layer was determined by an ellipsometer (Film Sense FS-1). To evaluate the mobility and carrier concentration of the ITiO material, Hall measurements were performed using a Lake Shore M91 FastHall system. Furthermore, to obtain the carrier concentration, the planar carrier density obtained from the Hall measurement was divided by the thickness of the ITiO layer. Table S2 provides a summary of the ITiO material characterization results.

Table S2: ITiO thin film characterization

Material	Type	Thickness (nm)	Mobility ($cm^2/(V·s)$)	Carrier concentration ($1/cm^2$)*	Carrier concentration ($1/cm^3$)**
ITiO	N	14	62.61	$172.42×10^{12}$	$1.23×10^{20}$

* Carrier concentration measurement obtained from hall measurement.
** Calculate carrier concentration per unit volume by dividing the measured planar carrier density by the thickness of the ITiO layer.

IV. C-V and I-V Characterization of the ITiO-Gated MOSCAP Si-MRM

Fig. S2(a) illustrates the cross-sectional view of the ITiO-gated MOSCAP Si-MRM. To characterize the electrical properties of the ITiO-gated MOSCAP Si-MRM, the gate bias on ITiO varied while Si was grounded. As depicted in Fig. S2(b), the experimental capacitance of the device was measured as a function of the gate bias. When the device was biased at -1.9 V for modulation, the capacitance was found to be 500 fF. This capacitance corresponds to a dielectric constant (κ) of 12, calculated based on the 62.5% active region of the microring. However, as shown in Fig. S2(a), the device includes a 500 nm wide Si slab attached to the inner ring. Although this slab increases the device's total capacitance, it does not contribute to the effective index modulation, which will be discussed later (Supplementary Information V). To accurately calculate the dynamic power consumption of the ITiO-gated MOSCAP Si-MRM, it is necessary to eliminate such parasitic capacitance. It can be determined by subtracting the contribution of the slab from the total capacitance, resulting in 333 fF. Using the formula ($CV^2/4$), the dynamic power consumption can be further calculated to be 53 fJ/bit. On the other hand, the static power consumption of the ITiO-gated MOSCAP Si-MRM is primarily determined by the leakage current (Fig. S2(c)). When the device is biased at -1.9V, the leakage current is measured to be 495 pA. The E-O tuning efficiency of 116 pm/V is employed to calculate the static power consumption, resulting in a value of 4.27 nW/nm.

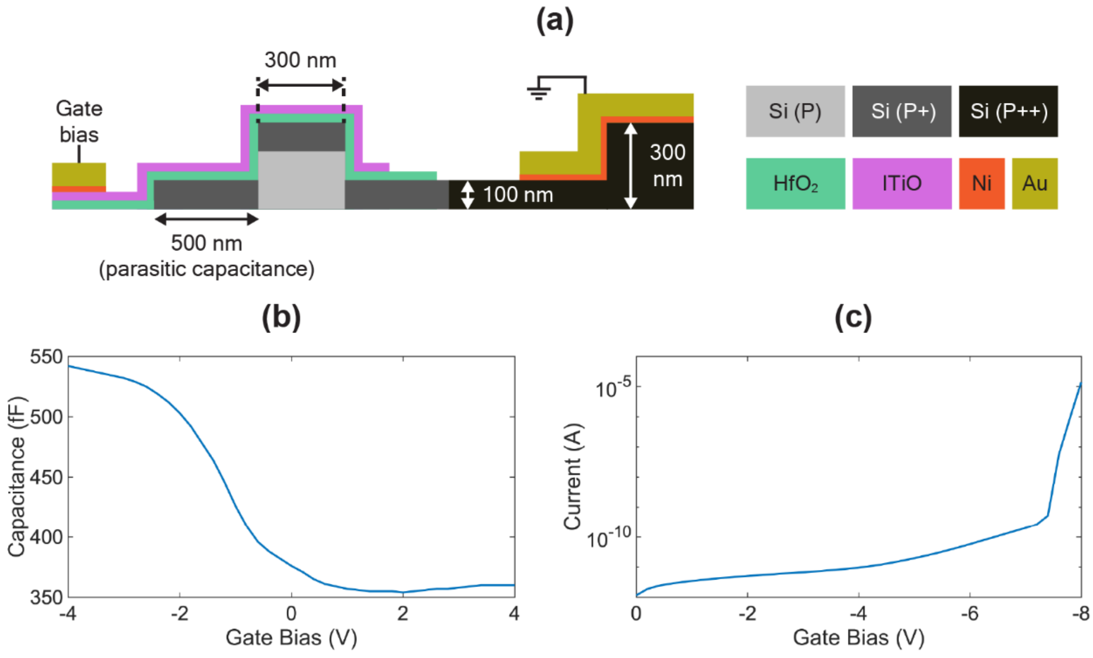

Fig. S2: Electrical characterization of the ITiO-gated MOSCAP Si-MRM with ITiO/HfO_2/Si MOSCAP. (a) the cross-sectional view in the active region of the ITiO-gated MOSCAP Si-MRM. (b) Capacitance-Voltage (C-V) Curve and (c) Current-Voltage (I-V) Curve.

V. Critical Coupling Condition of ITiO-Gated MOSCAP Si-MRMs

The ITiO-gated MOSCAP Si-MRMs with varying waveguide coupling gaps were fabricated on the same chip. Fig. 4(a) and Fig. S3(a) experimentally compare different coupling conditions. With a 200 nm gap, the device was near the critical coupling condition, resulting in deeper resonant dips as demonstrated in Fig. 4(a). Under this condition, the device exhibited a Q-factor of 4600 at 0V and an E-O efficiency of 117 pm/V. This configuration enables the device to achieve sub-volt modulation with 0.8 V_{pp}, as depicted in Fig. 4(c).

On the other hand, when the gap increases to 225 nm, the device was under-coupling, leading to shallower resonant dips as shown in Fig. S3(a). In this scenario, the device achieved a slightly higher Q-factor of 4700 at 0V due to reduced coupling losses and an E-O efficiency of 112 pm/V. However, even with similar Q-factor and E-O efficiency compared to the device operating near critical coupling, this device required a modulation voltage exceeding 1V_{pp}, as illustrated in Fig. S3(b) due to the shallower resonant dips. Therefore, it is evident that the critical coupling condition plays a pivotal role in reducing the driving voltage.

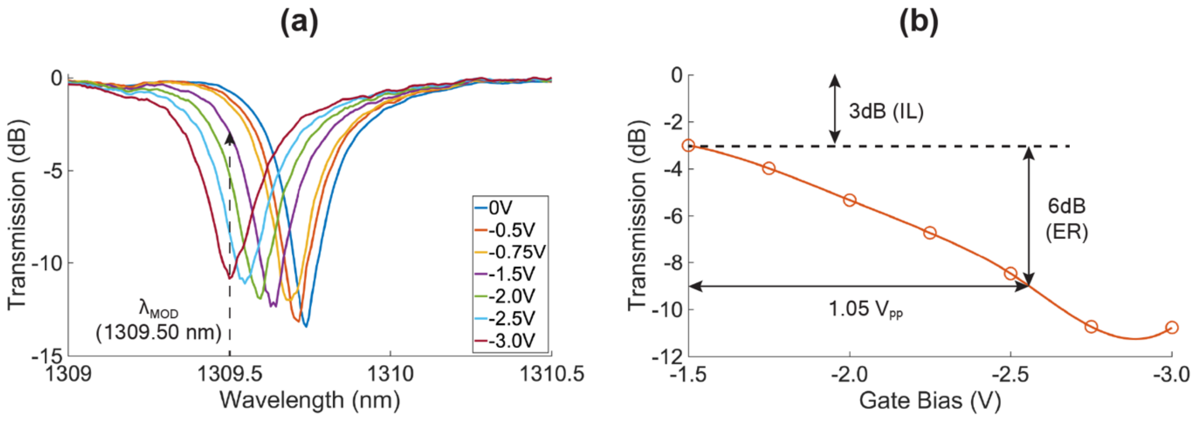

Fig. S3: DC characterization of ITiO-gated MOSCAP Si-MRM with the under-coupling condition. (a) Normalized transmission spectra with different gate biases. (b) Transmission at λ_{MOD} (1309.50 nm) with respect to the gate voltage.

VI. Analysis of Index Modulation Contribution in ITiO-Gated MOSCAP Si-MRM

In Fig. 2(b), the optical mode in the ring waveguide undergoes a shift towards the ring's outer edge due to the bending. This shift leads to varying degrees of overlap between the optical mode and different sides of the ring waveguide, affecting the effective index modulation (Δn_{eff}). To analyze the contributions of each side of the ITiO-gated MOSCAP Si-MRM to Δn_{eff}, the ITiO coverage region is divided into four sections, as shown in Fig. S4: (1) the top of the ring, (2) the outer sidewall, (3) the inner sidewall, and (4) the slab. Table S3 presents a comparison of the simulated effective index modulation per applied bias ($\partial n_{eff}/\partial V$) for each section. Narrowing the waveguide increases the overlap with the sidewalls, leading to a significant $\partial n_{eff}/\partial V$ on both sidewalls (sections 2 and 3). Additionally, due to the optical mode shifting towards the outer edge of the ring, section 2 exhibits a stronger $\partial n_{eff}/\partial V$ compared to section 3. Since the optical mode is primarily confined within the ring, the top of the ring (section 1) still contributes a small extent to $\partial n_{eff}/\partial V$. On the other hand, the contribution of $\partial n_{eff}/\partial V$ from the slab (section 4) is nearly negligible.

While sections 1 and 4 collectively contribute approximately 10% of the total $\partial n_{eff}/\partial V$, they account for 42.9% of the total capacitance. In contrast, sections 2 and 3 contribute about 90% of the total $\partial n_{eff}/\partial V$ while only contributing 57.1% of the total capacitance. As results, sections 1 and 4, particularly section 4, behave more like parasitic capacitance.

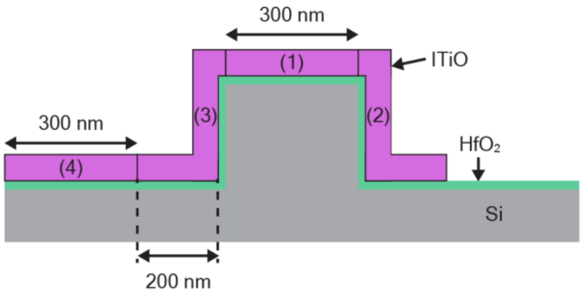

Fig. S4: Illustration for the division of ITiO coverage sections in the MRM waveguide. The MRM waveguide includes (1) the top side of the ring, (2) outer ring sidewall with 200 nm slab, (3) inner ring sidewall with 200 nm slab, and (4) 300 nm slab only.

Table S3: Comparison of simulated effective index modulation per applied bias among the different sections

Material	(1) Top	(2) Outer sidewall	(3) Inner sidewall	(4) Slab
$\partial n_{eff} / \partial V$ (1/V)	0.46×10^{-4}	2.49×10^{-4}	1.90×10^{-4}	0.03×10^{-4}
Percentage of modulation	9.43 %	51.03 %	38.93 %	0.61 %

VII. E-O Bandwidth Simulation Setup

Fig. S5 illustrates the simulation setup for the E-O bandwidth analysis. The simulation model follows the guidelines outlined on the Ansys/Optics website[10]. The MRM consists of a coupling gap (waveguide coupler), a passive region of the ring (WGD_1), an active region of the ring (WGD_2) with optical modulation (OM). Initially biased at -1.5V, the MRM operates in the accumulation regime of the MOSCAP.

WGD_1 exhibits a waveguide loss of 21.87 dB/cm, covering 30% of the ring, while WGD_2 has a waveguide loss of 111.27 dB/cm with 70% coverage. This MRM achieves Q-factors of approximately 5600. The MRM's RC bandwidth, functioning as the low pass filter (LPF), is configured to be 40.61 GHz.

The continuous wave laser (CWL) serves as the optical input with a wavelength of 1310.49 nm, chosen at the IL of 3 dB. The step source (STEP) inputs a small signal with an amplitude of 0.1 V as a perturbation. The optical oscilloscope (OOSC) receives the output signal, which undergoes a derivative to obtain the impulse response and then undergoes a Fourier transform to determine the E-O bandwidth. This E-O bandwidth encompasses both the electrical bandwidth (excluding the source impedance) and the optical bandwidth.

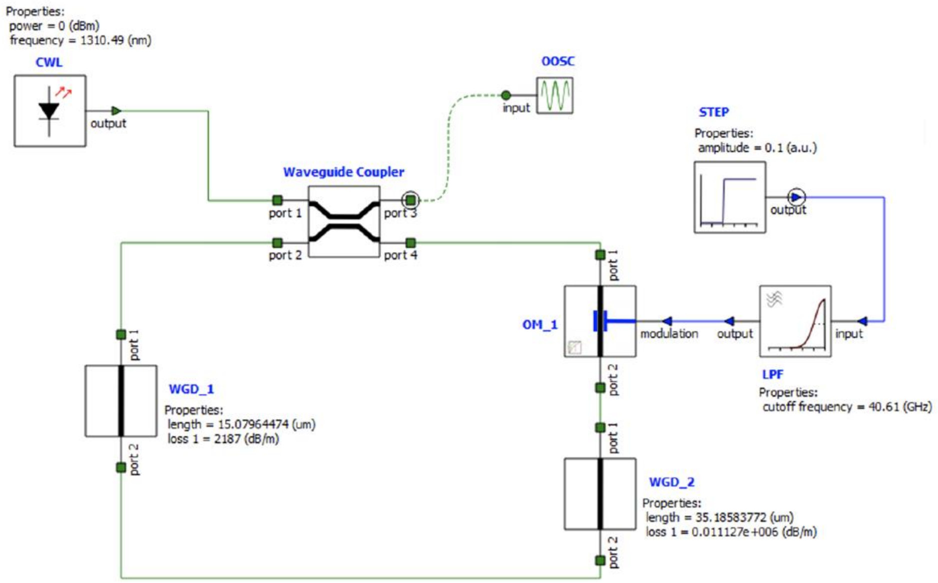

Fig. S5: E-O bandwidth simulation setup.

Supplementary References

1. Timurdogan, E. *et al.* An ultralow power athermal silicon modulator. *Nat. Commun.* **5**, 4008 (2014).

2. Sakib, M. *et al.* A high-speed micro-ring modulator for next generation energy-efficient optical networks beyond 100 Gbaud. in *CLEO* SF1C.3 (2021).

3. Zhang, Y. *et al.* 240 Gb/s optical transmission based on an ultrafast silicon microring modulator. *Photonics Res.* **10**, 1127–1133 (2022).

4. Campenhout, J. Van *et al.* Low-voltage, low-loss, multi-Gb/s silicon micro-ring modulator based on a MOS capacitor. in *OFC* OM2E.4 (2012).

5. Gevorgyan, H., Khilo, A., Wade, M. T., Stojanović, V. M. & Popović, M. A. Miniature, highly sensitive MOSCAP ring modulators in co-optimized electronic-photonic CMOS. *Photon. Res.* **10**, A1–A7 (2022).

6. Zhang, W. *et al.* Harnessing plasma absorption in silicon MOS ring modulators. *Nat. Photonics* **17**, 273–279 (2023).

7. Liang, D. *et al.* An Energy-Efficient and Bandwidth-Scalable DWDM Heterogeneous Silicon Photonics Integration Platform. *IEEE J. Sel. Top. Quantum Electron.* **28**, 1–19 (2022).

8. Kong, D., Liu, Y., Ding, Y., Hu, H. & Luan, C. *Ultrahigh-modulation-efficiency graphene-silicon micro-ring modulator*. (2023). doi:10.21203/rs.3.rs-2921645/v1.

9. Eppenberger, M. *et al.* Resonant plasmonic micro-racetrack modulators with high bandwidth and high temperature tolerance. *Nat. Photonics* **17**, 360–367 (2023).

10. Anysis/Optics. Ring modulator time domain (INTERCONNECT). https://optics.ansys.com/hc/en-us/articles/360042329094-Ring-modulator-time-domain-INTERCONNECT-.
