# Supplementary figures and images for: Sub-volt high-speed silicon MOSCAP microring modulator driven by high-mobility conductive oxide
Source: Nat Commun. 2024 Jan 27;15:826. doi: 10.1038/s41467-024-45130-4 (PMC10821938; doi:10.1038/s41467-024-45130-4)

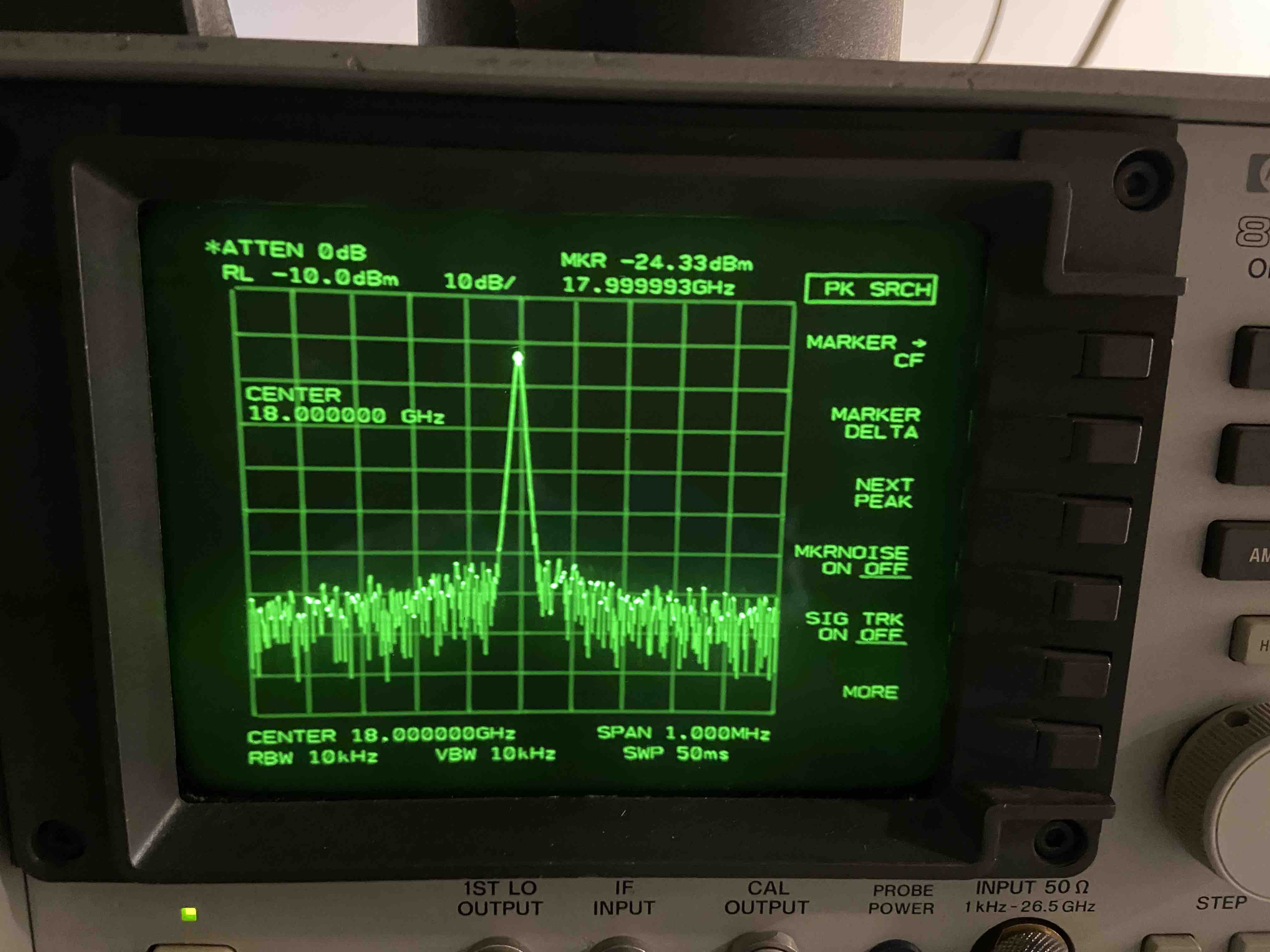

Supplement: Supplementary file 3 — Source Data [file 41467_2024_45130_MOESM3_ESM.zip › Data source/Fig.5_EO response/18GHz.jpg]

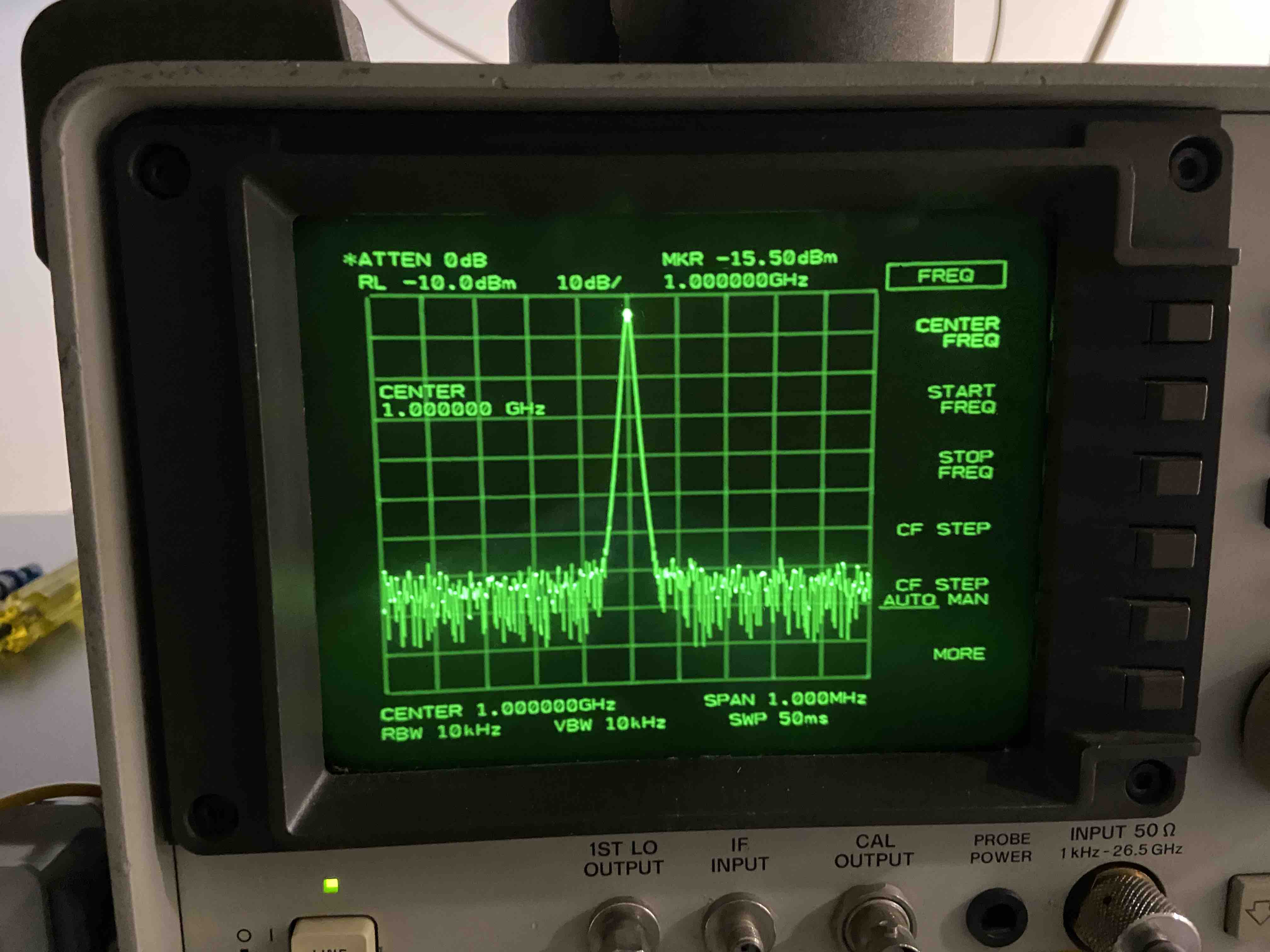

Supplement: Supplementary file 3 — Source Data [file 41467_2024_45130_MOESM3_ESM.zip › Data source/Fig.5_EO response/1GHz.jpg]

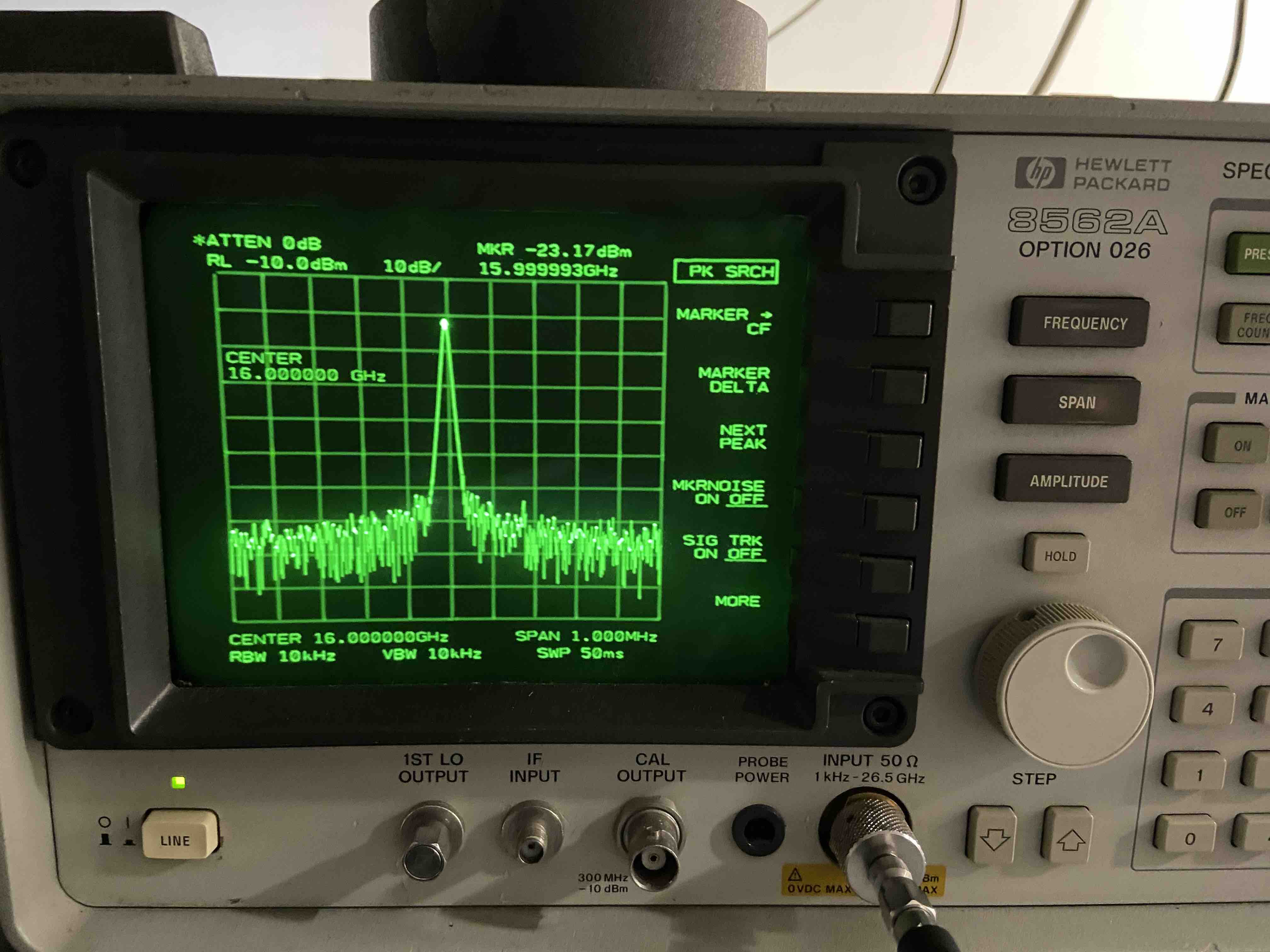

Supplement: Supplementary file 3 — Source Data [file 41467_2024_45130_MOESM3_ESM.zip › Data source/Fig.5_EO response/16GHz.jpg]

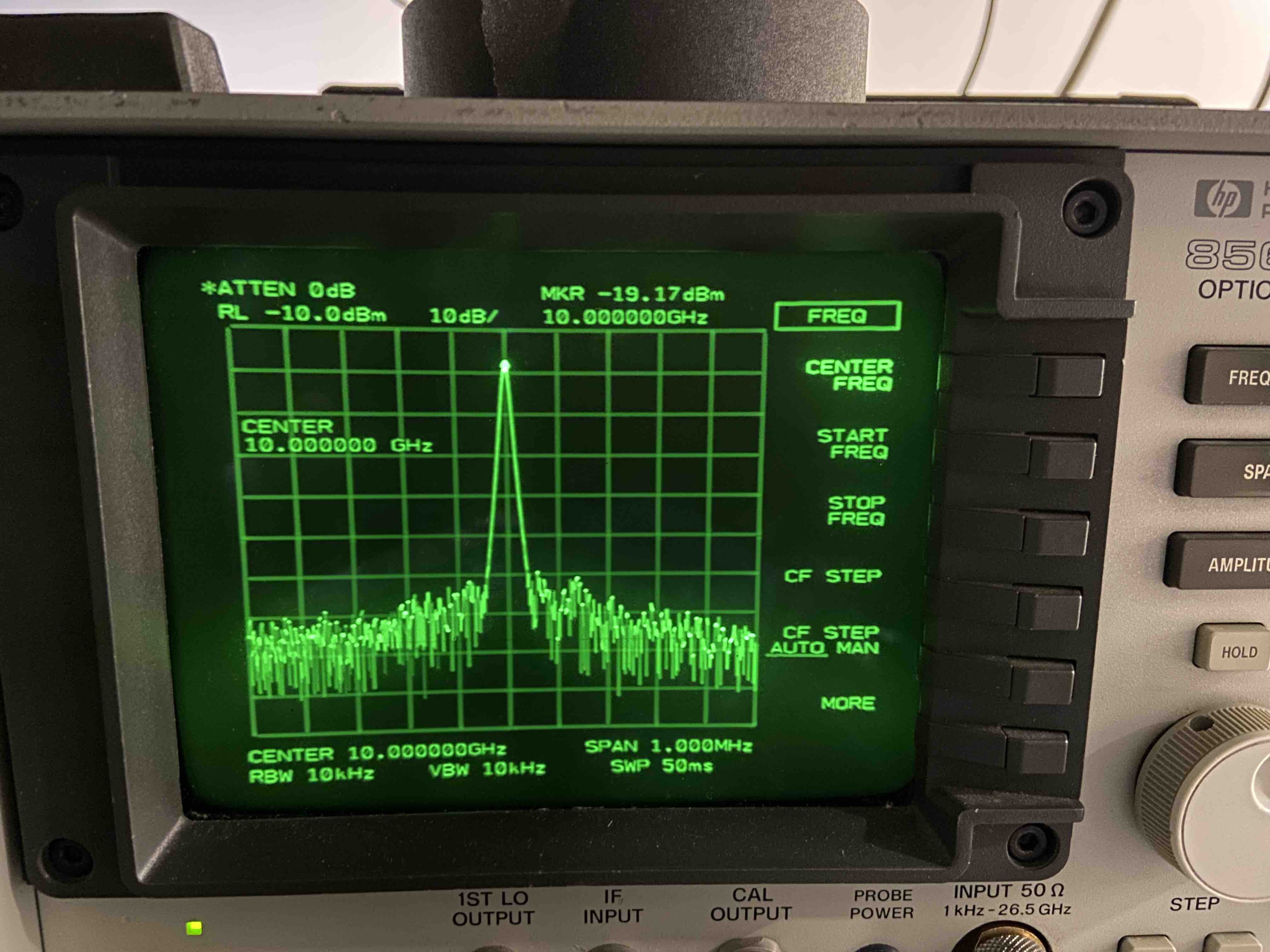

Supplement: Supplementary file 3 — Source Data [file 41467_2024_45130_MOESM3_ESM.zip › Data source/Fig.5_EO response/10GHz.jpg]

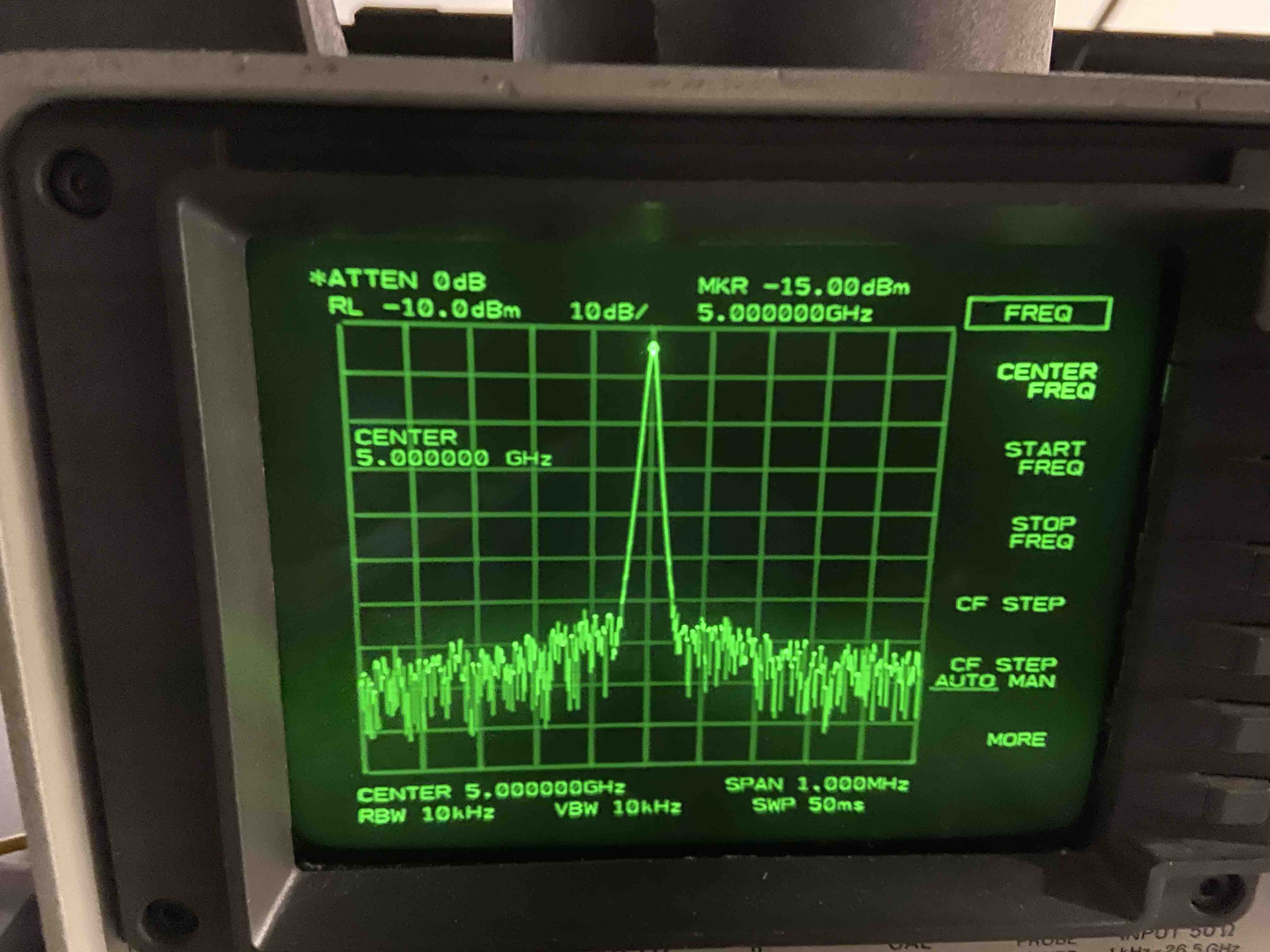

Supplement: Supplementary file 3 — Source Data [file 41467_2024_45130_MOESM3_ESM.zip › Data source/Fig.5_EO response/5GHz.jpg]

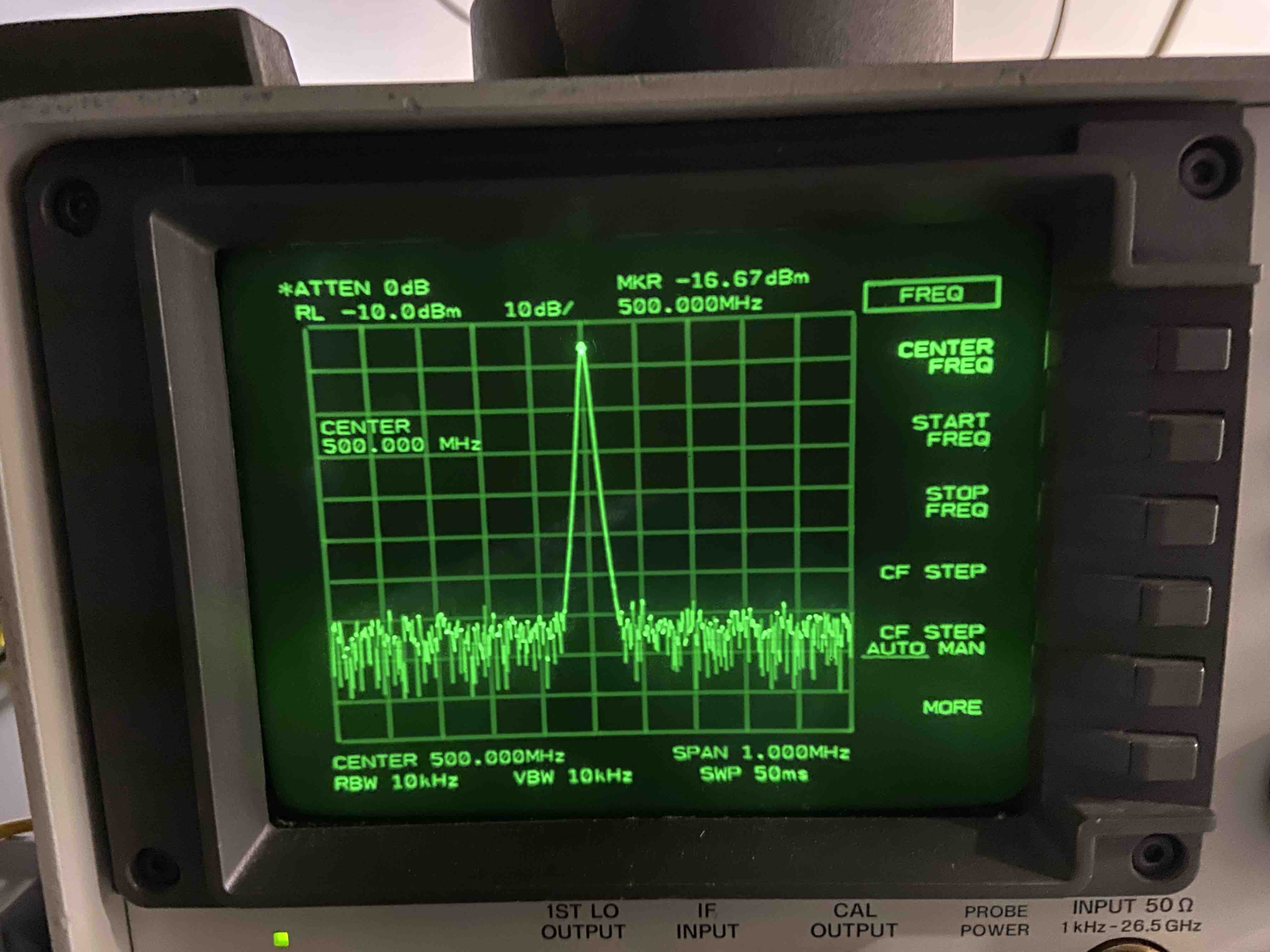

Supplement: Supplementary file 3 — Source Data [file 41467_2024_45130_MOESM3_ESM.zip › Data source/Fig.5_EO response/0.5GHz.jpg]

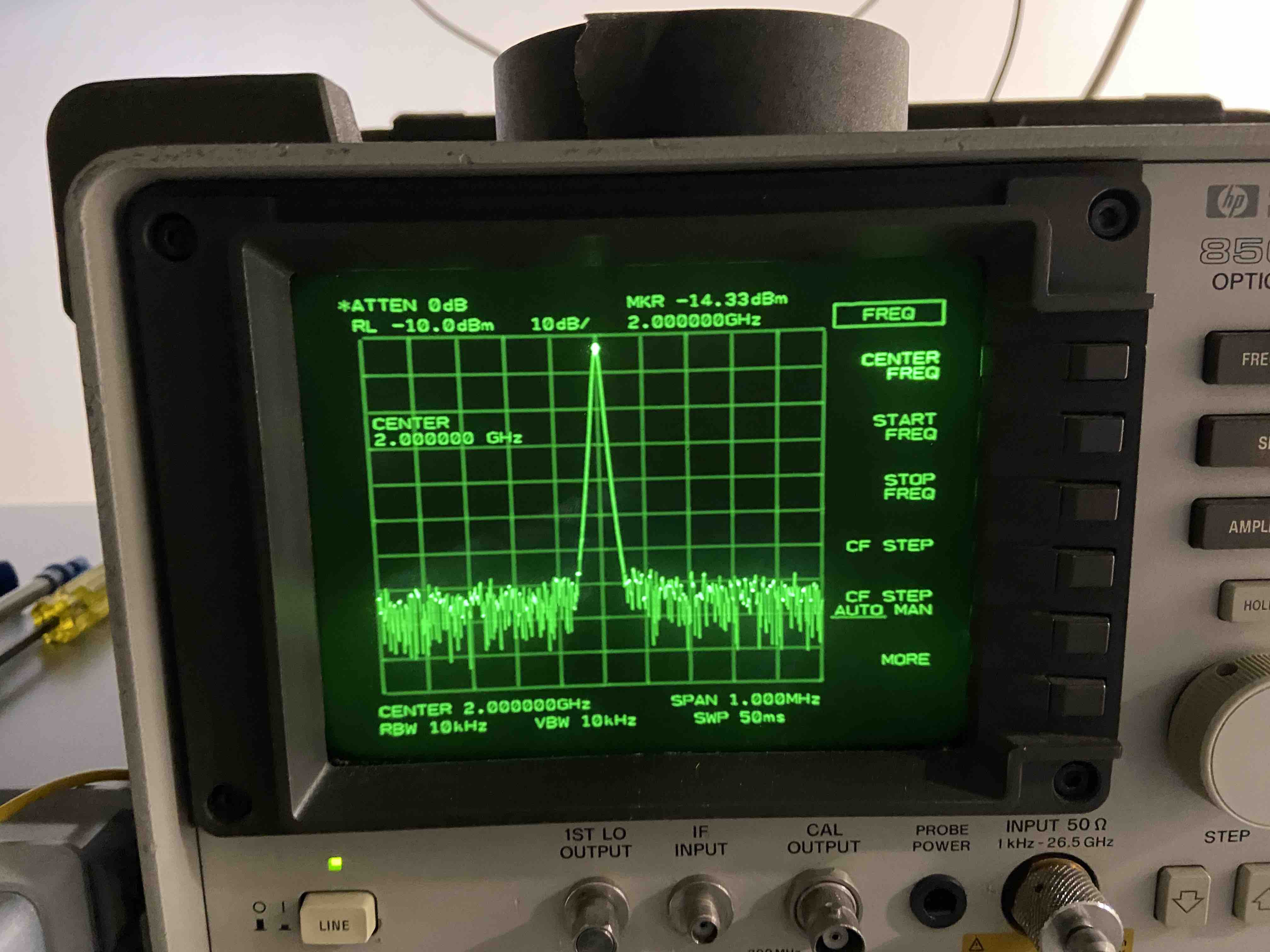

Supplement: Supplementary file 3 — Source Data [file 41467_2024_45130_MOESM3_ESM.zip › Data source/Fig.5_EO response/2GHz.jpg]

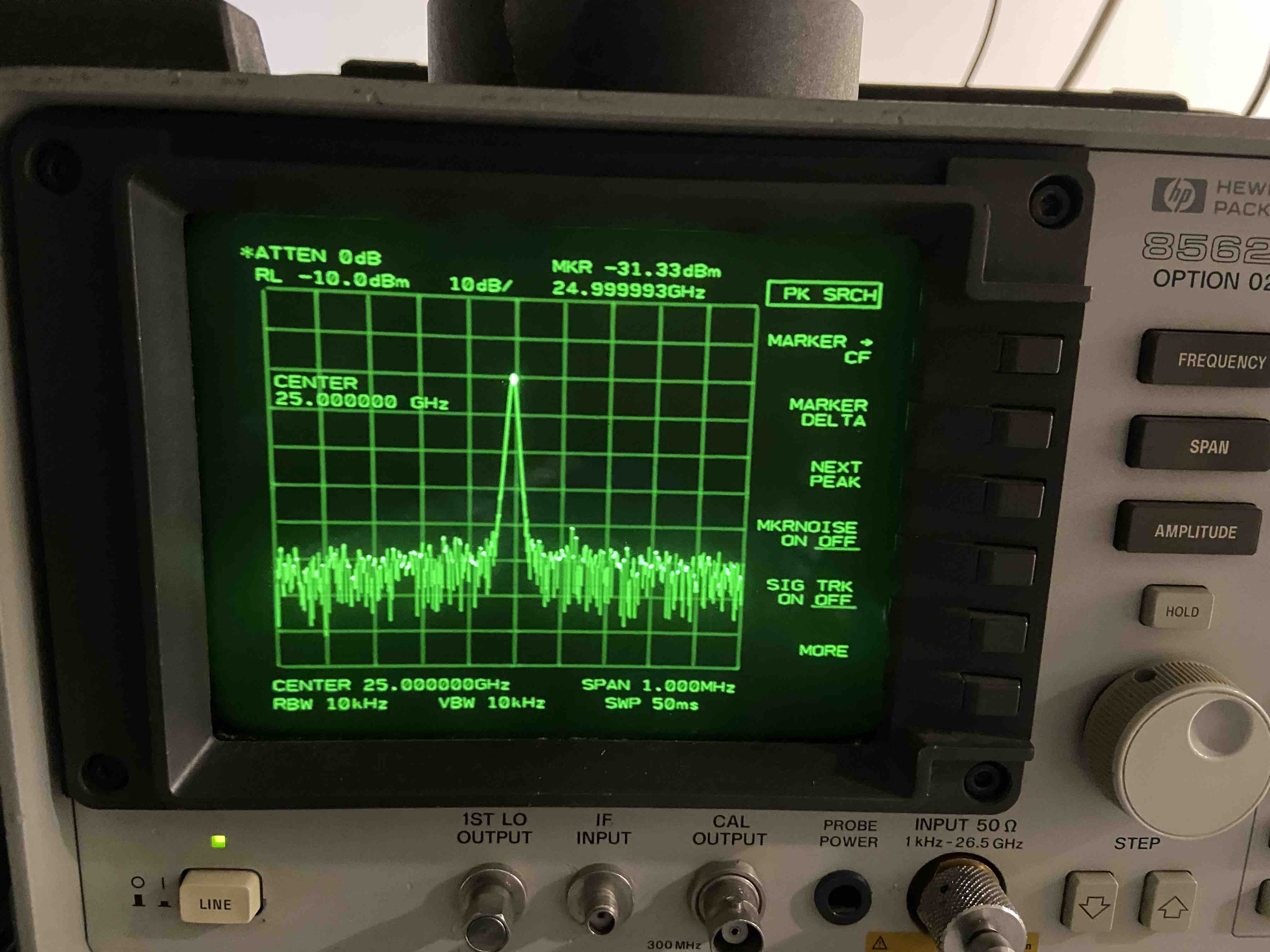

Supplement: Supplementary file 3 — Source Data [file 41467_2024_45130_MOESM3_ESM.zip › Data source/Fig.5_EO response/25GHz.jpg]

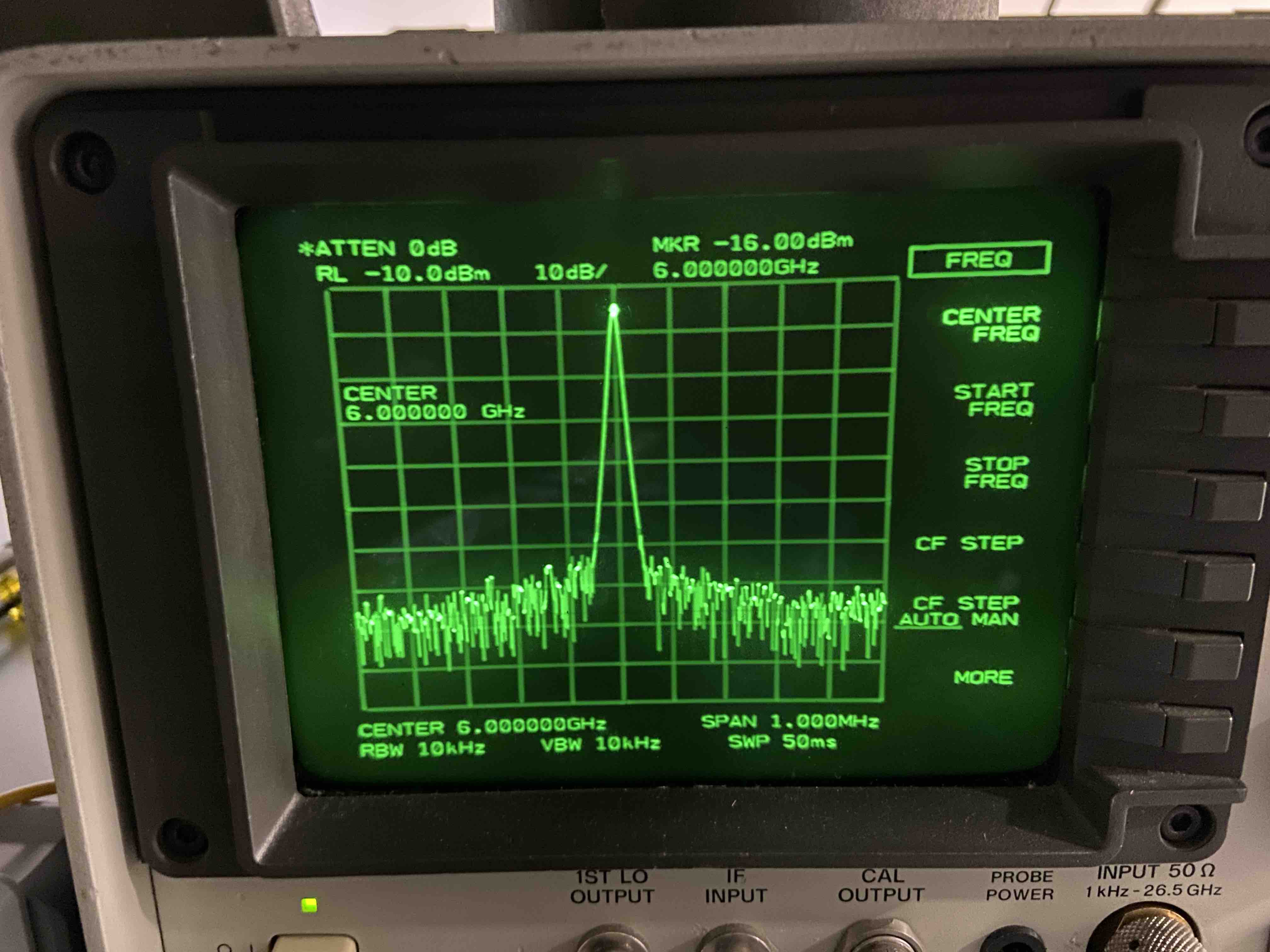

Supplement: Supplementary file 3 — Source Data [file 41467_2024_45130_MOESM3_ESM.zip › Data source/Fig.5_EO response/6GHz.jpg]

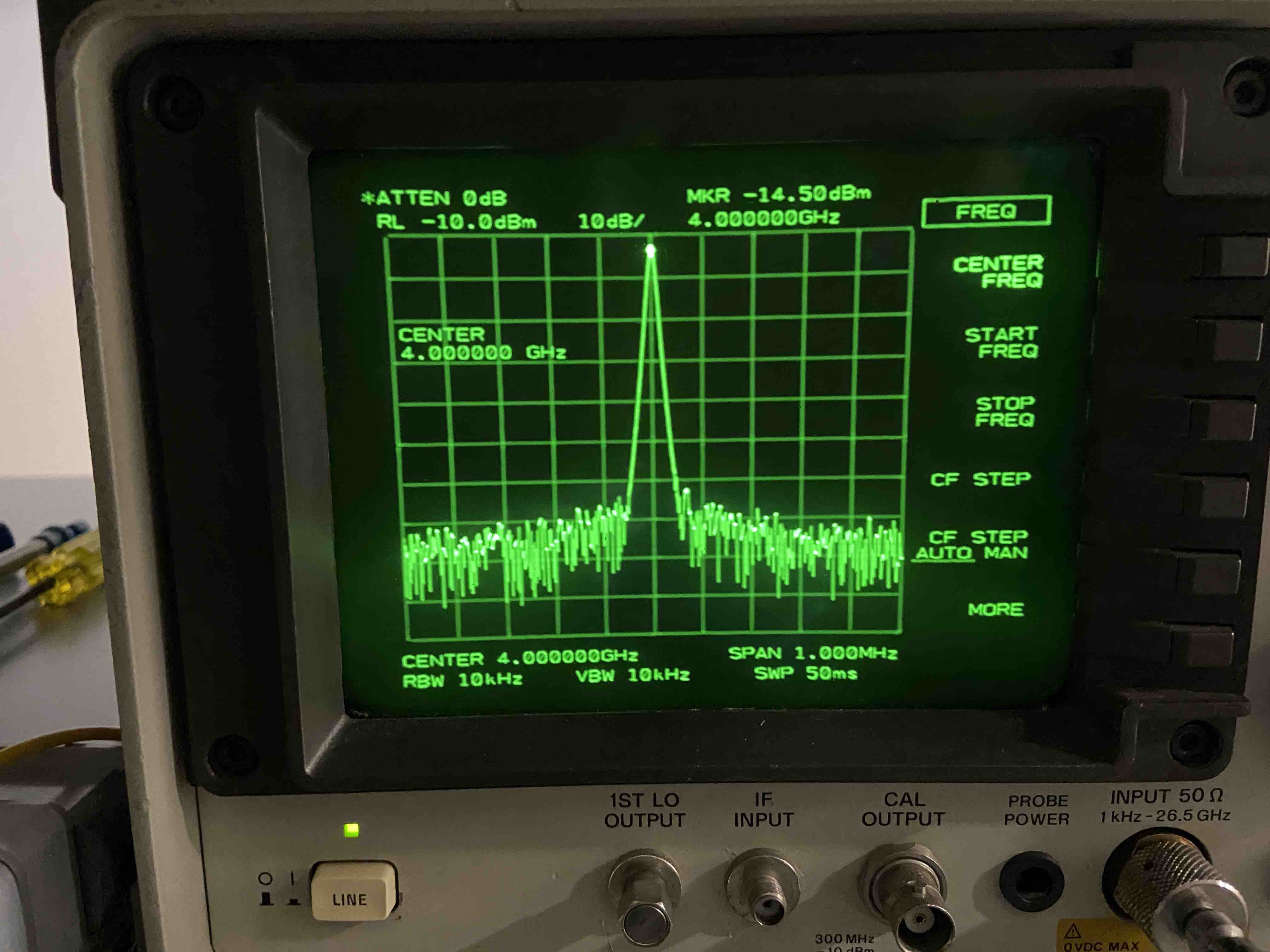

Supplement: Supplementary file 3 — Source Data [file 41467_2024_45130_MOESM3_ESM.zip › Data source/Fig.5_EO response/4GHz.jpg]

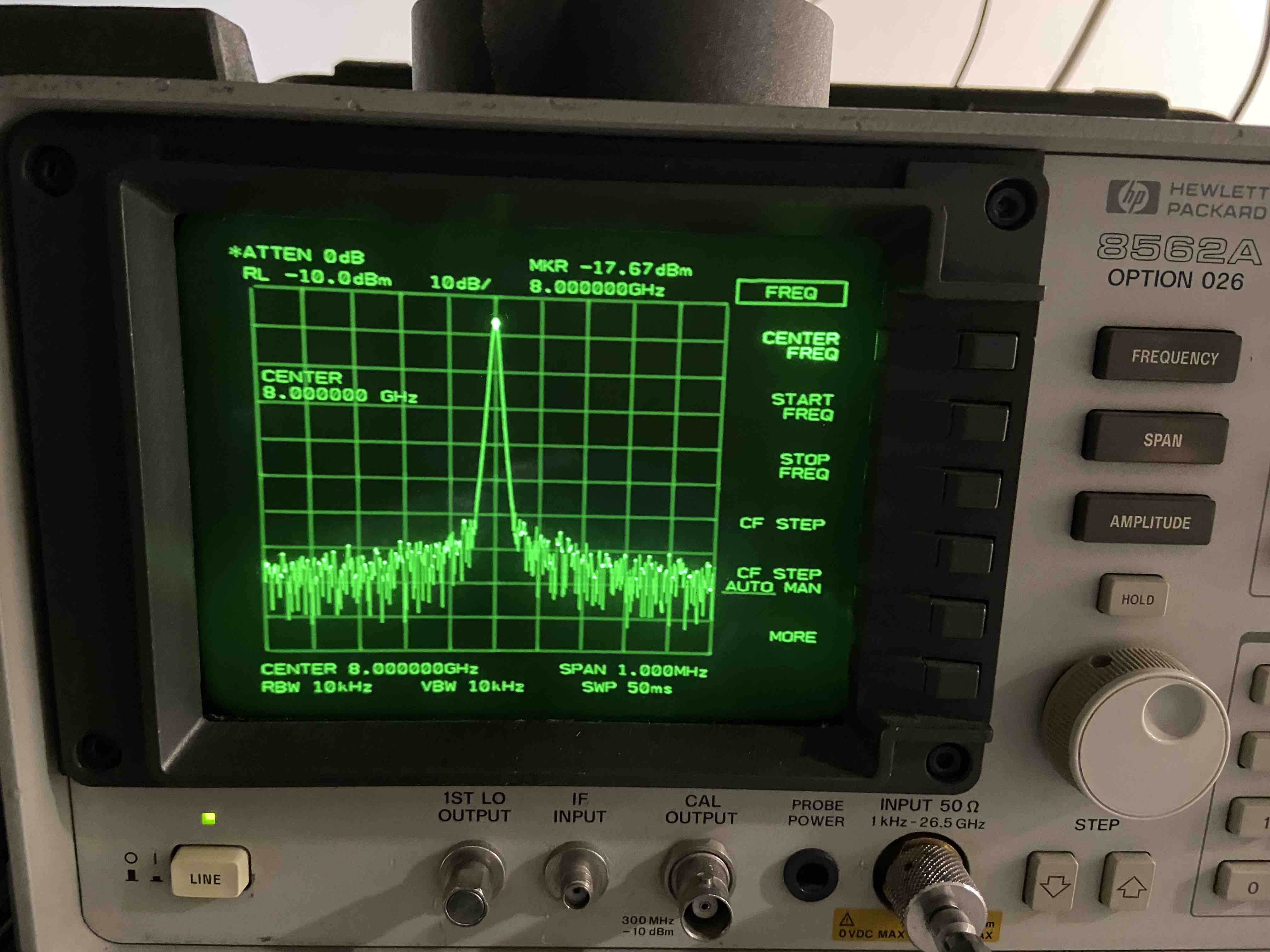

Supplement: Supplementary file 3 — Source Data [file 41467_2024_45130_MOESM3_ESM.zip › Data source/Fig.5_EO response/8GHz.jpg]

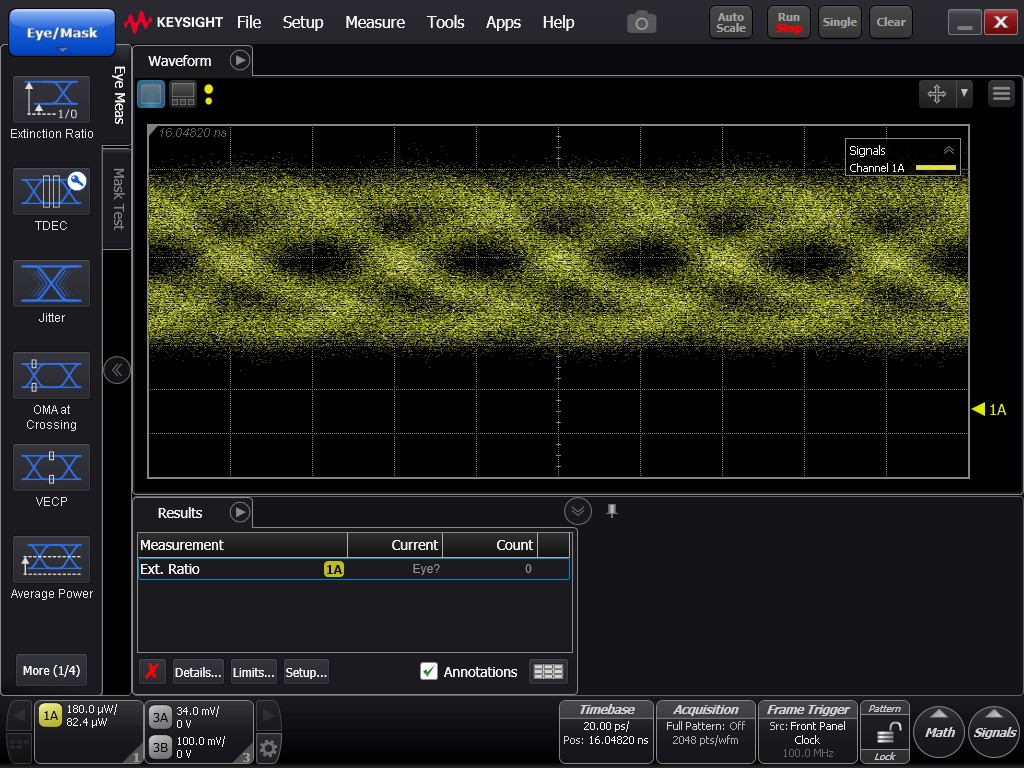

Supplement: Supplementary file 3 — Source Data [file 41467_2024_45130_MOESM3_ESM.zip › Data source/Fig.6_eye/1.75V_25G.jpg]

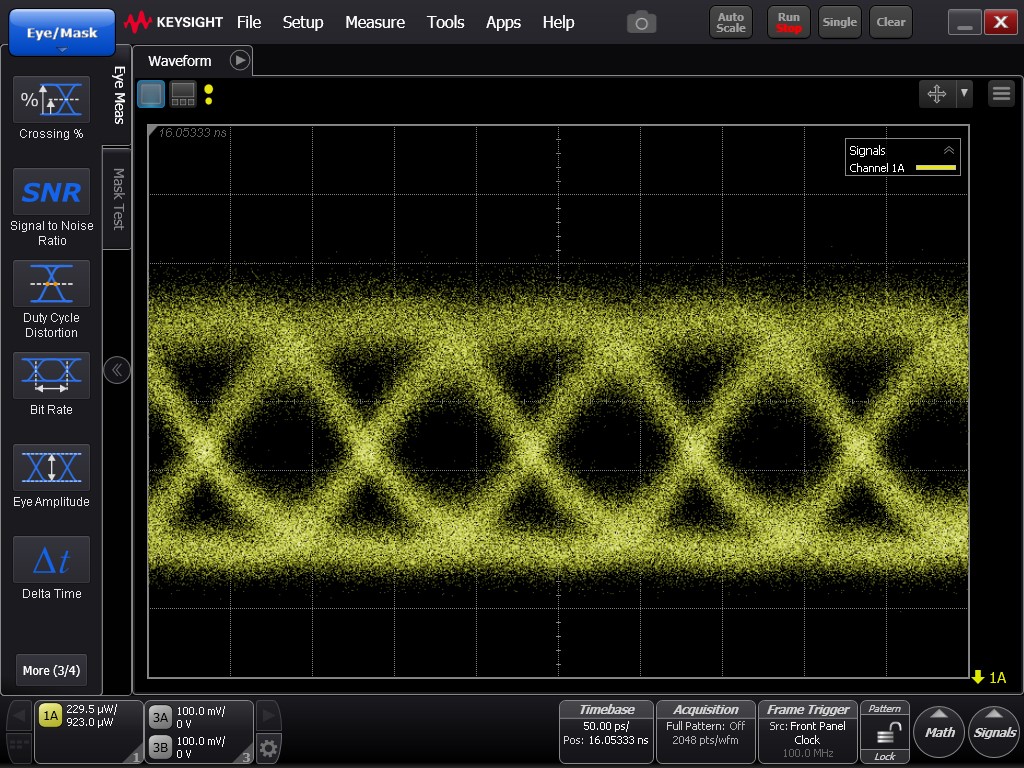

Supplement: Supplementary file 3 — Source Data [file 41467_2024_45130_MOESM3_ESM.zip › Data source/Fig.6_eye/0.8V_10G.jpg]

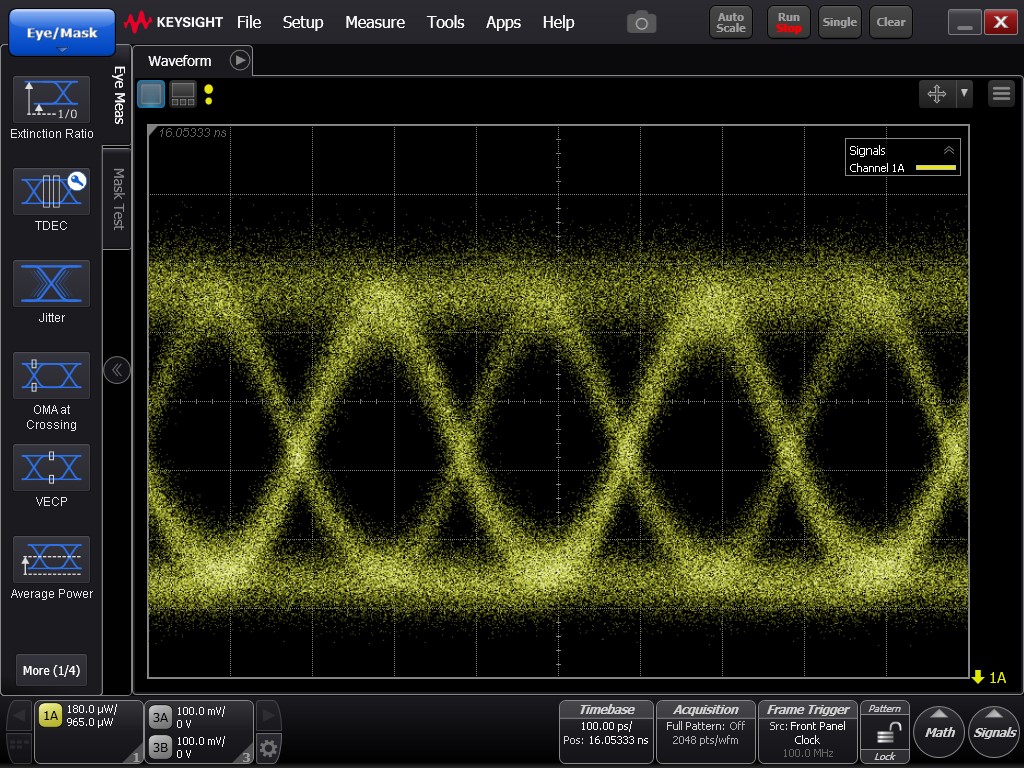

Supplement: Supplementary file 3 — Source Data [file 41467_2024_45130_MOESM3_ESM.zip › Data source/Fig.6_eye/0.8V_5G.jpg]

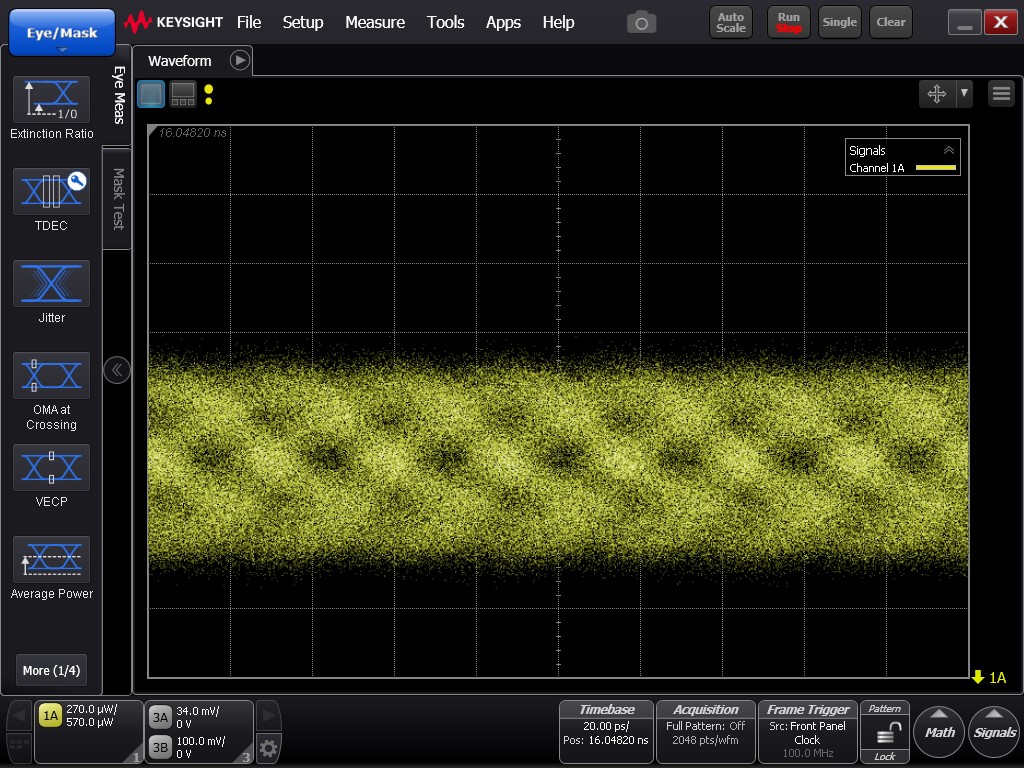

Supplement: Supplementary file 3 — Source Data [file 41467_2024_45130_MOESM3_ESM.zip › Data source/Fig.6_eye/1.75V_35G.jpg]

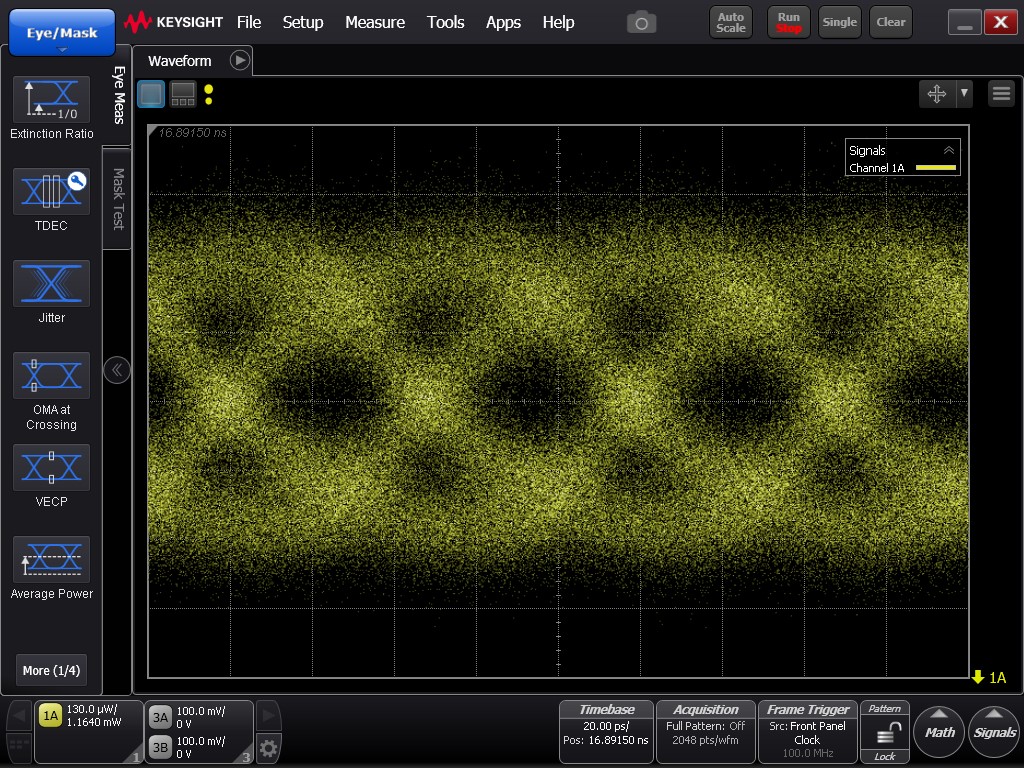

Supplement: Supplementary file 3 — Source Data [file 41467_2024_45130_MOESM3_ESM.zip › Data source/Fig.6_eye/0.8V_20G.jpg]

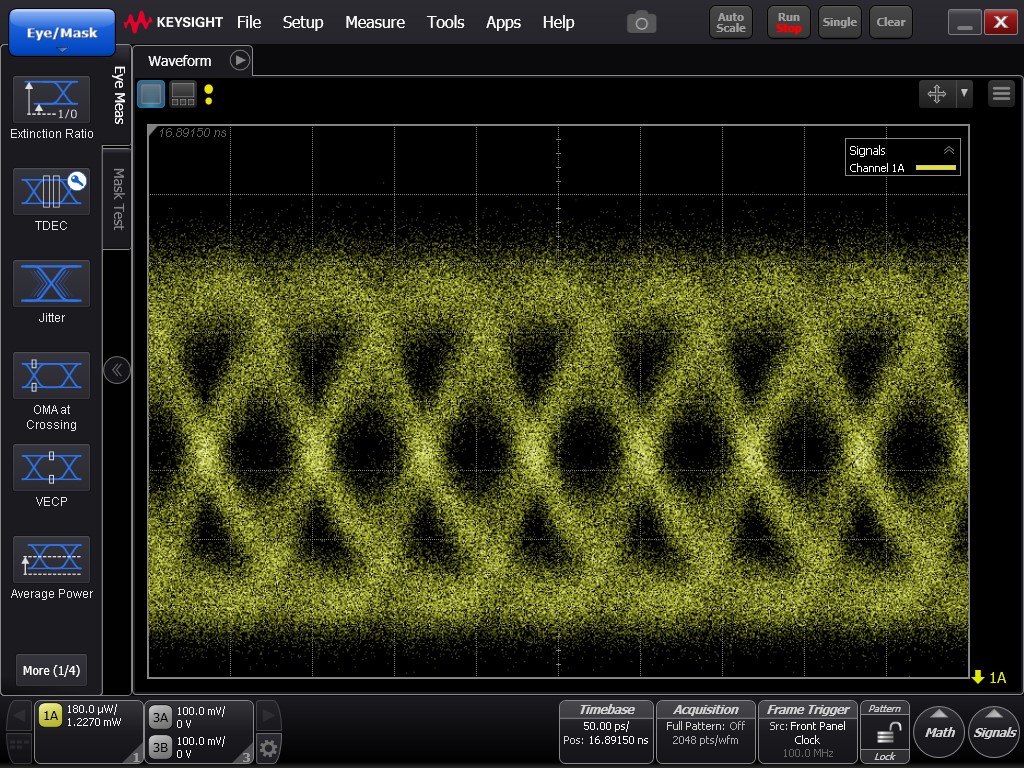

Supplement: Supplementary file 3 — Source Data [file 41467_2024_45130_MOESM3_ESM.zip › Data source/Fig.6_eye/0.8V_15G.jpg]

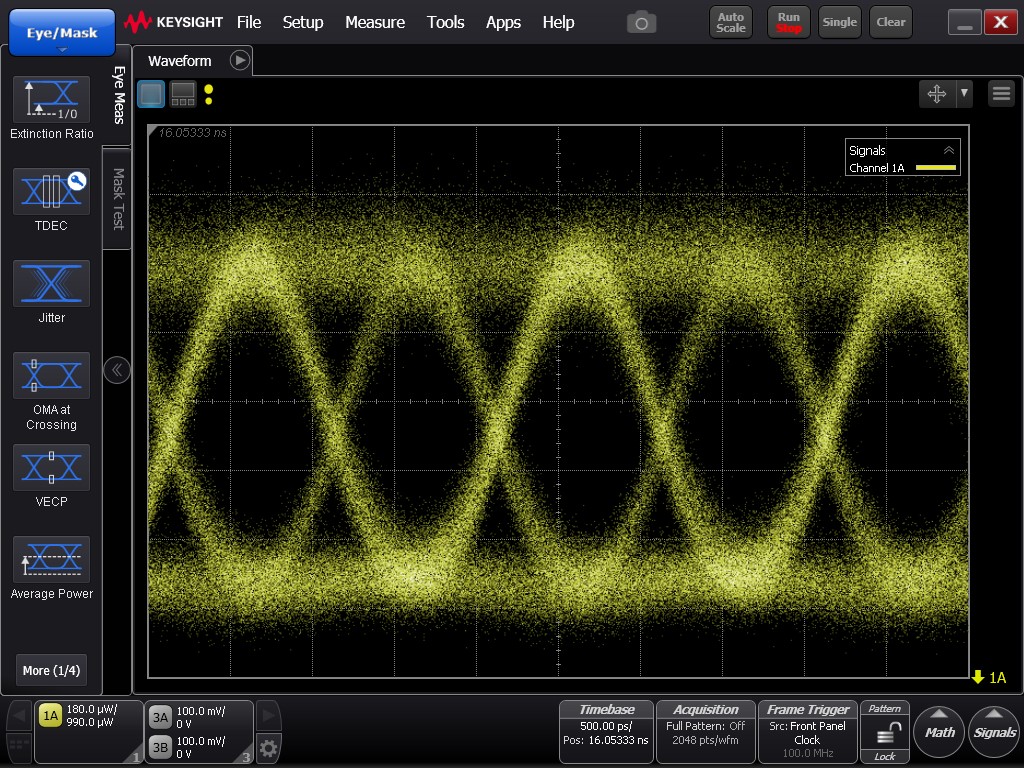

Supplement: Supplementary file 3 — Source Data [file 41467_2024_45130_MOESM3_ESM.zip › Data source/Fig.6_eye/0.8V_1G.jpg]

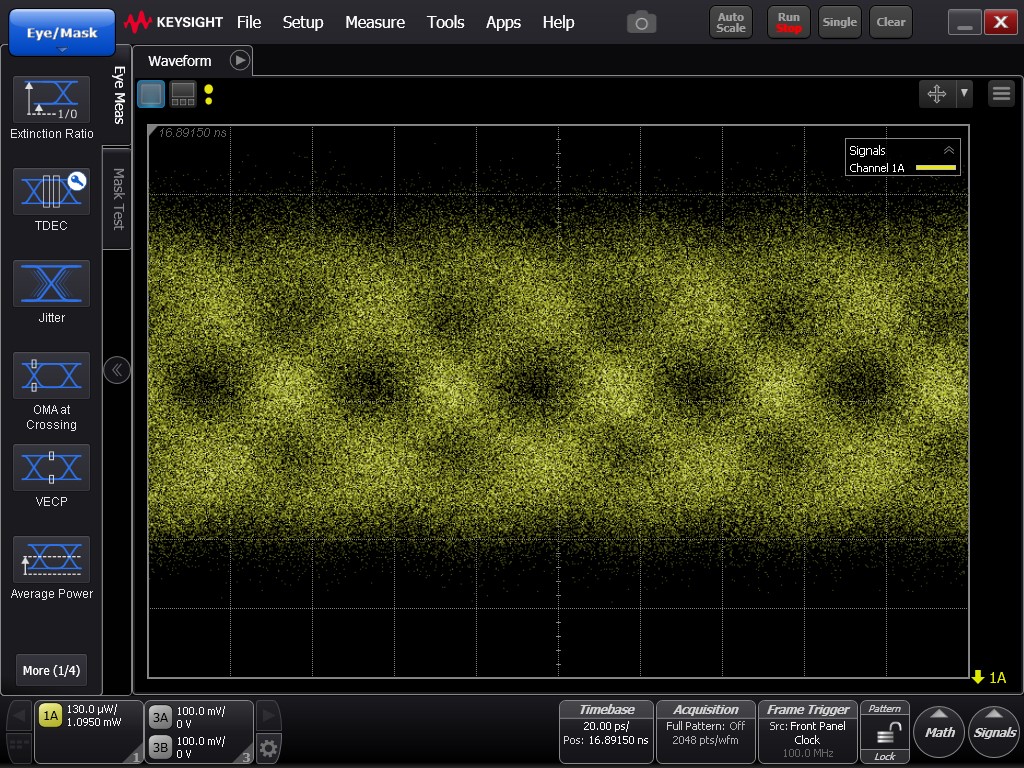

Supplement: Supplementary file 3 — Source Data [file 41467_2024_45130_MOESM3_ESM.zip › Data source/Fig.6_eye/0.8V_25G.jpg]

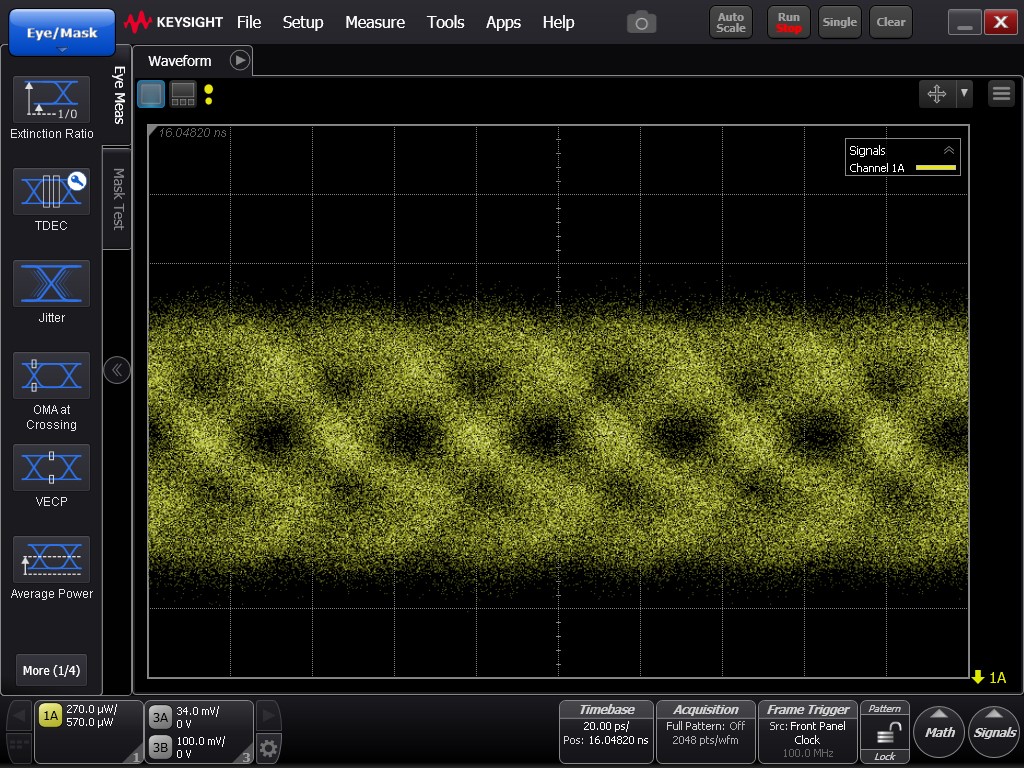

Supplement: Supplementary file 3 — Source Data [file 41467_2024_45130_MOESM3_ESM.zip › Data source/Fig.6_eye/1.75V_30G.jpg]
